# Elimination of Hepatitis B: Is It a Mission Possible?

**DOI:** 10.1186/s12916-017-0820-x

**Published:** 2017-03-15

**Authors:** Tai-Chung Tseng, Jia-Horng Kao

**Affiliations:** 1 Department of Internal Medicine, National Taiwan University Hospital Jinshan Branch, New Taipei City, Taiwan; 2 Division of Gastroenterology, Department of Internal Medicine, National Taiwan University Hospital, Taipei, Taiwan; 3 Graduate Institute of Clinical Medicine, National Taiwan University College of Medicine, 1 Chang-Te St., Taipei, 10002 Taiwan; 4 Hepatitis Research Center, National Taiwan University Hospital, Taipei, Taiwan; 5 Department of Medical Research, National Taiwan University Hospital, Taipei, Taiwan

**Keywords:** HBV, Chronic hepatitis B, HBsAg, Immunotherapy

## Abstract

Chronic hepatitis B virus (HBV) infection is a global public health issue. Although the disease cannot be cured effectively, disease management has been improved over the past decade. The introduction of potent nucleos(t)ide analogues (NAs) to suppress viral replication represented a giant leap in the control of this disease. It has been shown that tenofovir treatment, a potent NA, complements current immunoprophylaxis to diminish mother-to-infant transmission in pregnant women with a high viral load. For patients with chronic HBV infection, quantitative hepatitis B surface antigen is a useful tool to define inactive carriers and to guide antiviral therapy. Quantification of HBV mutants is also useful in predicting long-term outcomes more precisely than ever. The next challenge is how to achieve an HBV cure; although immunotherapy is a promising strategy, the current results from two clinical trials using therapeutic vaccines to induce HBV-specific immune response in patients with chronic HBV infection are disappointing. In the coming years, we are expecting to see a combination of therapeutic agents with various modes of action to complete the mission of HBV elimination.

## Background

Chronic hepatitis B virus (HBV) infection continues to be a major global public health issue despite the availability of effective HBV vaccines for over three decades. Recent data estimated that more than 240 million individuals worldwide are positive for hepatitis B surface antigen (HBsAg) [1]. Several viral, host, and environmental factors have been identified in subjects who are at increased risk of developing liver cirrhosis, hepatic decompensation, and hepatocellular carcinoma (HCC) [2].

Although HBV has been discovered for more than half a century, a cure for chronic hepatitis B (CHB) remains a challenging task [3]. Currently approved antiviral treatments for CHB include nucleos(t)ide analogues (NAs) and interferon. NAs effectively suppress HBV replication to undetectable levels through inhibition of viral reverse transcriptase. However, rebound of viremia frequently occurs after discontinuation of NA, primarily because of the persistence of the active transcriptional template of HBV covalently closed circular DNA (cccDNA). In contrast, interferon is known to have a dual effect – direct suppression of viral replication and indirect enhancement of host immunity against the virus. Nevertheless, the overall viral response rate of 30% to interferon is far from satisfactory. In this minireview, recent advances of HBV management and strategies to eliminate HBV will be summarized and discussed.

## Management of patients with CHB

### Role of quantitative HBsAg in predicting HCC development and treatment response

The application of qualitative HBsAg in the clinical management of CHB patients has been increasingly adopted [4]. It is generally believed that serum HBV DNA level is a major driver of disease progression in CHB patients [5]. In particular, patients with serum HBV DNA levels ≥ 2000 IU/mL at study entry have an increased risk of developing HCC over time. In contrast, those with HBV DNA levels < 2000 IU/mL are usually designated as low viral load patients. Data from two independent cohorts from Taiwan consistently showed that serum HBsAg levels of 1000 IU/mL could stratify different HCC risks in patients with low viral loads [6, 7]. In addition to HCC risks, a lower serum HBsAg level (<1000 vs. > 1000 IU/mL) has also been shown as an indicator of lower risk of viral relapse, hepatitis B e antigen (HBeAg)-negative hepatitis, and cirrhosis development in CHB patients [8–11]. These emerging data infer that we may define minimal-risk HBV carriers by combining low viral load (<2000 IU/mL) and low HBsAg level (<1000 IU/mL) [11]. Another clinical usefulness of quantitative HBsAg is to guide physicians regarding cessation of antiviral therapy. Serum HBsAg levels > 20,000 IU/mL at week 24 of treatment are used to predict non-responders in HBeAg-positive patients receiving 12-month pegylated interferon therapy [12]. In addition, quantitative HBsAg may predict who can maintain a sustained viral response after stopping NA treatment [13–15]. Although the reported HBsAg cutoff levels ranged from 10 to 150 IU/mL, the data consistently showed that the lower the HBsAg level, the lower the risk of viral and clinical relapse. Further large-scale prospective studies are needed to define a reliable HBsAg cutoff level for identification of CHB patients who can cease NA treatment safely.

### Role of quantitative viral mutants in predicting different clinical outcomes

Quantification of HBV mutants has also become a useful tool for the management of CHB infection. HBV is the smallest human DNA virus and viral mutants develop during the course of persistent infection because of the spontaneous error of viral reverse transcription. Two common HBV mutants, precore stop codon (G1896A) and basal core promoter (BCP; A1762T/G1764A) mutations, can respectively abolish and reduce the production of HBeAg [16]. The presence of these viral mutants has been shown to be associated with disease progression to HCC [17, 18]. However, these viral mutants were determined using qualitative population sequencing, and therefore the data provided limited information to correlate with clinical profiles [17, 18]. With advances in biotechnology, quantification of these viral mutants is now possible (Table 1). Quantification of precore/BCP can serve as a predictor of HBeAg seroconversion either spontaneously or induced by interferon-based treatment [19, 20]. The percentage of BCP mutants can be used to predict clinical outcomes such as the development of liver cirrhosis [21]. Furthermore, the ultimate tool for viral genome research, next-generation sequencing, has been recently introduced into HBV research, being able to determine the minor variant even at the level of 1%. Recent data using next-generation sequencing to determine viral variants has been shown to predict HBsAg loss in HBV carriers receiving 4-year tenofovir (TDF) treatment, a potent NA [22]. However, these findings need to be validated by subsequent larger studies. Thus, quantification of HBV mutants provides viral information in greater detail, allowing clinicians to implement precision medicine in the foreseeable future.

**Table 1:** Quantification of HBV variants and its clinical application

	Nie et al. [19]	Yang et al. [20]	Tseng et al. [21]	Bayliss et al. [22]
Quantification assay	Selective inhibitory polymerase chain reaction	Pyrosequencing	Pyrosequencing	Next generation sequencing
Target region	PC (G1896A) & BCP (A1762T/G1764A) mutants	PC (G1896A) & BCP (A1762T/G1764A) mutants	BCP mutants (A1762T/G1764A)	HBV whole genome
Sensitivity to detect minor strains	<1%	10%	10%	<1%
Enrolled patients	18 HBeAg-positive patients	203 HBeAg-positive patients receiving interferon-based treatment	151 HBeAg-negative patients with a median follow-up period of 9 years.	157 HBeAg-positive patients receiving 4-year tenofovir treatment
Main finding	Levels of PC and BCP mutants may predict the time of HBeAg seroconversion	Quantitative analysis of PC and BCP mutants can predict interferon-induced HBeAg seroconversion	A higher percentage of BCP mutant is associated with higher risks of cirrhosis development	Detectable BCP or PC mutants are associated with a lower probability of HBsAg loss during tenofovir therapy.

*Note*

*PC* precore stop codon, *BCP* basal core promoter, *HBeAg* hepatitis B e antigen, *HBsAg* hepatitis B surface antigen

## Strategies to eliminate HBV

### Combination of TDF and immunoprophylaxis to minimize mother-to-child transmission (MTCT) of HBV

Interruption of HBV transmission routes is the most effective way to reduce the global burden of HBV infection. The combination of hepatitis B immunoglobulin and hepatitis B vaccine as immunoprophylaxis in newborns has been shown to reduce the rate of MTCT from 90 to 10% [23]. However, immunoprophylaxis implementation has been reported to have a failure rate of 10–30% in infants born to mothers with an HBV DNA level of more than 200,000 IU/mL [23]. To overcome the gap, a non-randomized study from Taiwan firstly showed that MTCT risk was reduced by TDF treatment in the third trimester of pregnancy in HBeAg-positive mothers with high viral loads [24]. The results were further reinforced by a randomized controlled study in China [25]. The per-protocol analysis showed that the transmission rate dropped from 7% in the control group to 0% in the TDF treatment group at postpartum week 28 (Table 2). Although a longer duration and larger number of participants are needed to evaluate the safety profile in infants, a short-term TDF therapy for pregnant mothers with HBV DNA levels > 200,000 IU/mL does minimize MTCT risk. Therefore, this will become the standard of care to prevent HBV transmission in this unique clinical setting.

**Table 2:** Summary of 2 clinical trials using tenofovir to reduce mother-to-infant transmission on top of standard immunoprophylaxis

Study	Chen et al. [24]	Pan et al. [25]
Study design	Prospective non-randomized control trial	Prospective randomized control trial
No. of mother	TDF (*N* = 92), Control (*N* = 56)	TDF (*N* = 97), Control (*N* = 100)
Intervention	TDF vs. Control	TDF vs. Control
Time of intervention	30–32 weeks of gestation	30-32 weeks of gestation
Maternal viral load	≥20,000,000 IU/mL	≥200,000 IU/mL
Maternal HBeAg-positive rate	100%	100%
Mother-to-infant transmission rate	TDF (1.54%) vs. Control (10.71%), *P* = 0.0481*	Intention-to-treat analysis: TDF (5%) vs. Control (18%), *P* = 0.007** Per-protocol analysis: TDF (0%) vs. Control (7%), *P* = 0.01

*Note*

*TDF* tenofovir, *HBeAg* hepatitis B e antigen

* Defined by HBsAg positivity at 6 months postpartum

** Defined by serum HBV DNA level of more than 20 IU per milliliter (i.e., above the lower limit of detection) or HBsAg positivity at 28 weeks postpartum. Participants who lost to follow-up or who discontinued treatment were counted as having treatment failure

### The possible mission of functional cure for HBV

**Fig. 1:**
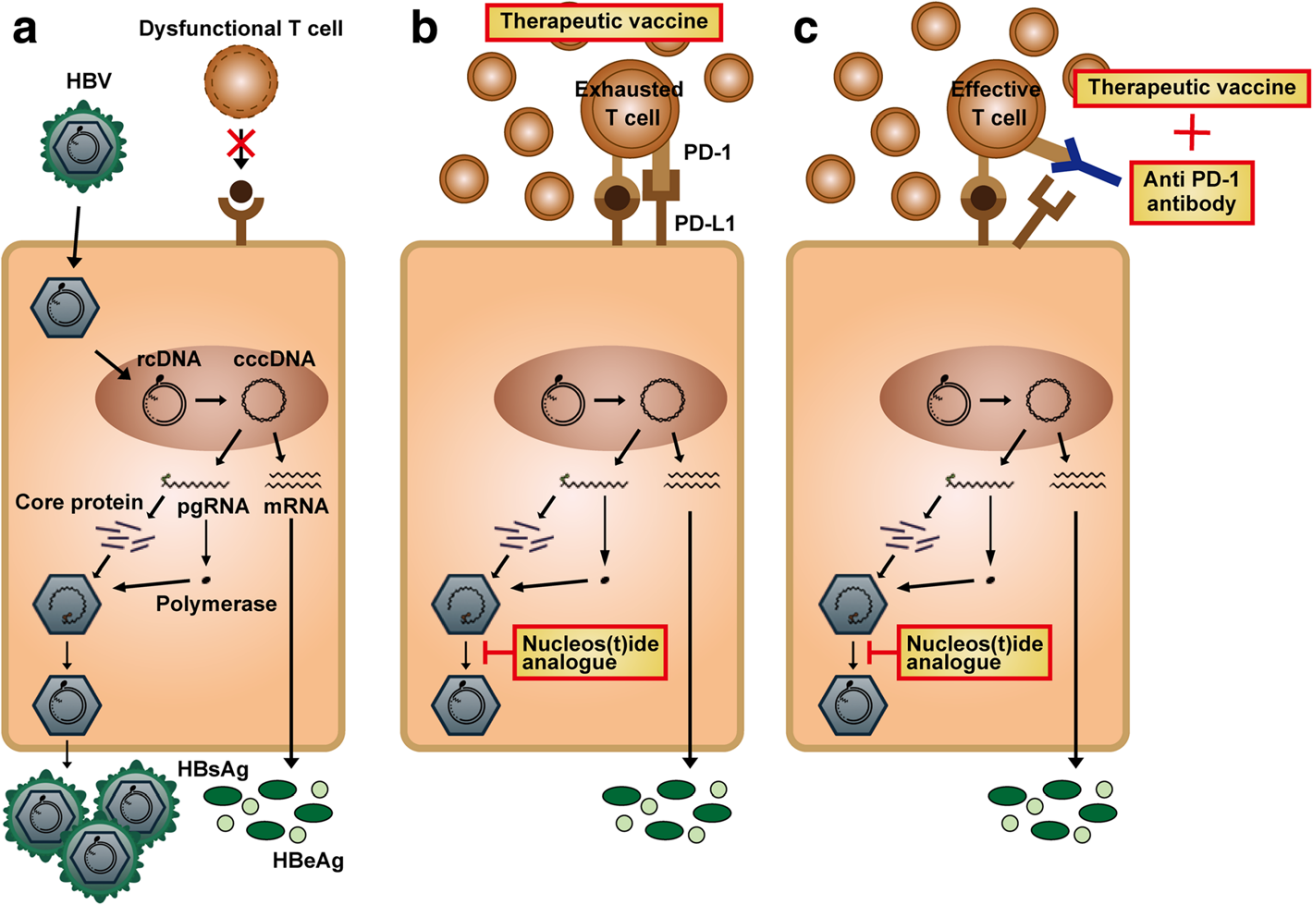
**a** Patients with chronic hepatitis B are characterized by a high viral load and antigenaemia, as well as a small number of dysfunctional HBV-specific T-cells. **b** Failure of combining therapeutic vaccine and nucleos(t)ide analogue treatment could be attributed to T cell exhaustion induced by PD1 and PD-L1 engagement. **c** Combining anti-PD1, an immune checkpoint inhibitor, with strategy **b** may be a solution to cure chronic HBV infection

HBsAg loss is regarded as a functional cure for HBV and serves as an ideal treatment endpoint. Nevertheless, it is rare to achieve the ultimate goal using current treatment modalities. There are several novel strategies to clear HBsAg, including killing of HBV-infected hepatocytes via cytotoxic T cell (CTL)-induced immunotherapy as the most promising one. Although HBV-specific CTL response is vigorous and multi-specific during acute HBV infection, it is usually weak or even undetectable during the CHB stage [26]. An ideal immune-therapeutic strategy should combine profound suppression of viral replication to prevent uninfected hepatocytes from HBV infection and restoration of HBV-specific CTL response to clear the infected hepatocytes. The former goal can be achieved by existing NA treatment and the later one could be partially enhanced by therapeutic vaccines [27]. To date, two clinical trials have been performed using different therapeutic vaccines to treat CHB patients with a similar strategy, yet the results of both have been disappointing [28, 29]; neither of the two therapeutic vaccines could clear HBsAg more effectively compared to the control group. There are two issues that must be considered. Firstly, HBV-specific CTL function has been shown to be preserved in children and young adults, but not in older patients [30]. Since both studies enrolled older patients, HBV-specific CTL response may fail to be triggered by therapeutic vaccinations. Second, the immune tolerant effects of the liver microenvironment must be considered. HBV-specific CTL response can be induced in peripheral blood, but is rapidly exhausted after the initiation of cytotoxic effects against HBV-infected hepatocytes (Fig. 1a,b). Either of the above issues may have led to the failure of the two clinical trials. If the failure was caused by the engagement of PD-1 on T cells and PD-L1 on hepatocytes, which leads to the inhibition of T cell receptor-mediated lymphocyte proliferation and cytokine secretion, an immune checkpoint inhibitor, such as anti-PD1, could be considered as an addition to the therapeutic vaccine for the amplification of these effects (Fig. 1c). Indeed, the success of this combination strategy has been demonstrated in a woodchuck model [31]. Although the current results of immunotherapy in CHB treatment are unsatisfactory, it remains the most attractive method to clear the virus as long as the appropriate patient population can be selected and optimal study designs can be implemented.

## Perspectives

Potent NA treatment has improved the management of CHB infection over the past decade [32]. Prolonged NA treatment has halted disease progression and halved the HCC incidence in CHB patients who have already developed liver cirrhosis [33–35]. The next challenge is to achieve functional HBV cure and the development of new agents with various modes of action is urgently awaited. In the clinical practice, additional unmet needs should also be addressed. The first is to identify patients who require HCC surveillance. Previous studies have indicated that approximately 25–40% of patients with CHB infection will develop HCC in their lifetime, and therefore HCC surveillance is indicated [36]. Although prediction of HCC is difficult, it is possible to identify true inactive HBV carriers who are at the lowest risk of HCC development by combining multiple host, viral, and liver fibrosis markers. The current criteria to define inactive carriers include normal ALT level, negative HBeAg, HBV DNA < 2000 IU/mL, and HBsAg < 1000 IU/mL; their HCC risk is comparable to that of the normal population [6]. The possibility of further reduction of HCC risks by including biomarkers, such as a liver biomarker indicating early liver fibrosis stage, along with the current criteria should be explored.

The second issue to be addressed is whether HCC development could be prevented by earlier initiation of NA treatment in CHB patients. Most of the evidence of HCC risk reduction comes from prolonged NA treatment in patients with HBV-related liver cirrhosis [33–35]. However, all studies show that HCC still develops during long-term NA therapy. This fact may suggest HCC development is inevitable once advanced liver fibrosis has occurred. Further evidence has been provided by the recent finding that the integration of the HBV genome into the host chromosome, which is considered as an oncogenic event, could be detected at the early stage of chronic infection [37]. Collectively, it is conceivable to achieve an HCC-free era through early initiation of NA therapy before emergence of significant fibrosis or massive viral genome integration. This may be implemented into practice by widening the therapeutic target of HBV carriers and earlier identification using more comprehensive screening. This strategy may provide an alternative method to eliminate HCC risk before curing HBV. However, this concept needs to be proved by clinical studies.

## Conclusions

Various measures, including universal hepatitis B vaccination and interruption of transmission routes, are required to reach the ultimate goal of global HBV eradication. Potent NA treatment has remarkably improved the prevention of MTCT and management of CHB. We are expecting to see less disease progression and less HCC development in CHB patients following initiation of prolonged NA therapy. However, the current armamentarium does not completely clear HBsAg, and new combination treatment modalities are needed to achieve a functional HBV cure in the near future, hopefully by the year 2030 [38].

## Abbreviations

cccDNA: Covalently closed circular DNA
CHB: Chronic hepatitis B
CTL: Cytotoxic T cell
HBeAg: Hepatitis B e antigen
HBsAg: Hepatitis B surface antigen
HCC: Hepatocellular carcinoma
MTCT: Mother-to-child transmission
NA: Nucleos(t)ide analogue
pgRNA: pregenomic RNA
TDF: Tenofovir
